# Interaction of BSA with Ta_2_O_5_ Nanoparticles: The Effect of Polydopamine Pre-Coating

**DOI:** 10.3390/molecules31020241

**Published:** 2026-01-11

**Authors:** Ekaterina Koshevaya, Nikita Lifanovsky, Elena Shishmakova, Maksim Staltsov, Alexander Dubovik, Alexandr Belousov, Dmitry Kaluzhny, Vladimir Kuzmin, Vladimir Morozov, Maria Kolyvanova, Olga Dement’eva

**Affiliations:** 1 State Research Center—Burnazyan Federal Medical Biophysical Center, Federal Medical Biological Agency of the Russian Federation, 23 Marshala Novikova, Moscow 123182, Russia; belousovav@physics.msu.ru (A.B.); kolyvanova@physics.msu.ru (M.K.); 2 Emanuel Institute of Biochemical Physics, Russian Academy of Sciences, 4 Kosygina, Moscow 119334, Russia; lifanovskyns@yandex.ru (N.L.); adubovik@ineos.ac.ru (A.D.); vladimirkuzmin7@gmail.com (V.K.); morozov.v.n@mail.ru (V.M.); 3 National Research Nuclear University MEPhI, 31 Kashirskoye Shosse, Moscow 115409, Russia; m.staltsov@gmail.com; 4 Frumkin Institute of Physical Chemistry and Electrochemistry, Russian Academy of Sciences, 31-4 Leninsky Prospect, Moscow 119071, Russia; alena_shishmakova@mail.ru (E.S.); dema_ol@mail.ru (O.D.); 5 Engelhardt Institute of Molecular Biology, Russian Academy of Sciences, 32 Vavilova, Moscow 119991, Russia; uzhny@mail.ru

**Keywords:** tantalum oxide nanoparticles, polydopamine coating, bovine serum albumin, fluorescence spectroscopy, protein adsorption, colloidal stability

## Abstract

The modification of tantalum oxide (Ta_2_O_5_) nanoparticles (NPs) with biocompatible polymers is crucial for their biomedical use. Such modification can prolong NP circulation in the bloodstream by minimizing salt-induced aggregation and reducing nonspecific protein adsorption onto their surface. Understanding the features of polymer–NP interactions is a key issue in the fabrication of nanostructures with required characteristics. The present work aims to provide a comprehensive comparative study of bovine serum albumin (BSA) adsorption on bare and polydopamine (PDA)-coated Ta_2_O_5_ NPs. The synthesized NPs were characterized via transmission electron microscopy, Fourier transform infrared spectroscopy, dynamic light scattering, and zeta potential measurements. Fluorescence and circular dichroism spectroscopy were also employed for the first-time investigation of the interactions of Ta_2_O_5_ NPs and Ta_2_O_5_@PDA NPs with BSA. The results obtained show that PDA coating significantly enhances the protein-binding affinity. Time-resolved measurements revealed signatures of Förster resonance energy transfer, confirming complex formation between NPs and BSA. Moreover, colloidal stability tests in phosphate-buffered saline indicated that the presence of adsorbed BSA improves the dispersion stability of bare and PDA-coated Ta_2_O_5_ NPs. These findings advance the understanding of protein–NP interactions and highlight the potential of PDA coatings for designing stable and functional nanostructures for biomedical applications.

## 1. Introduction

Nowadays, a lot of drug forms based on nanoparticles (NPs) have either entered clinical practice or are undergoing clinical trials [1]. Among the variety of NPs being studied for medical purposes, those made of tantalum pentoxide (Ta_2_O_5_) attract special attention. This material is known as an inert, highly biocompatible, and radiopaque. Owing to its high chemical stability and minimal adverse effects, it seems to be an ideal material for coating surgical implants [2,3,4]. Furthermore, in the nanoparticulate form, tantalum oxide is promising for the development of drug-delivery systems [5], biosensors [6], contrast agents [7,8], and antitumor radiosensitizers [9,10].

One of the key limitations in the development of effective products based on NPs is the difficulty in predicting their behavior in vivo. Getting into the body presents a serious problem for NPs, since the environment there is far from “friendly” for them. For example, when administered intravenously, NPs encounter not only high ionic strength, molecular crowding, and whatever follows but also the immune defense system (for more information on opsonization, phagocytic attacks, etc., see [11,12]. In this regard, special attention should be paid to the physical or colloidal stability of NPs, as well as how to make them “stealthy” to the immune system, since, among others, these factors largely determine biodistribution, pharmacokinetics, and toxicity [13].

A promising strategy to ensure the stability of nanodrugs in physiological environments and prevent opsonization is the preliminary coating of NP surface with proteins [14,15,16]. These molecules may provide both steric and electrostatic stabilization to NPs due to their large number of charged groups. Among various proteins, bovine serum albumin (BSA) is one of the most commonly used for these purposes due to its abundance, high biocompatibility and non-toxicity to cells. However, BSA, very similar in structure and function to its human analog, is much cheaper and more available on the market. In principle, BSA molecules can be adsorbed onto the surface of metal oxide NPs through hydrogen bonding, electrostatic and hydrophobic interactions [17]. Also, to ensure effective binding of BSA molecules, a special functional layer may be formed on the NP surface. For this purpose, various polymers, such as, for example, polydopamine (PDA), can be used. PDA interacts with BSA through Michael addition, Schiff base reaction, and several non-covalent interactions [18,19], and, by the way, coating PDA NPs with BSA has been previously demonstrated as an effective strategy to prevent particle aggregation [20]. PDA also contains diverse functional groups, including catechol, amine, and imine, which enable it to form coatings on inorganic surfaces and provide a versatile platform for further modification.

It is especially important to note here that practically nothing is known about the interaction of Ta_2_O_5_ NPs with BSA. There are some works devoted to the adsorption of this protein on tantalum oxide films (for example, see [21]), but for its nanoparticulate form, there is an absolute lack of corresponding information. By the way, this problem concerns the NPs of many metal oxides. If many such works are known for metallic NPs (e.g., gold or silver), then only a few are known for those made, for example, of zinc, zirconium or cerium oxide (see [22,23]). From this point of view, the most studied among them, apparently, are iron and titanium oxide NPs (this is in good agreement with the data by Goodilin et al. [24]). Thus, the lack of systematic data on the interaction of metal oxide NPs with albumin and other serum proteins represents a serious challenge given how many factors influence this process (see in detail in [25]) and, therefore, how difficult it is to extrapolate the known results.

In the present work, we studied, for the first time, the interaction of Ta_2_O_5_ NPs with BSA, tested the possibility of facilitating the adsorption of its molecules onto the particle surface by its pre-coating with PDA, and also carried out a preliminary assessment of how coating such NPs with protein affects their stability under conditions close to physiological ones, since like a number of other NPs, those made of tantalum oxide are unstable in electrolyte solutions with high ionic strength [26,27].

## 2. Results and Discussion

Tantalum oxide NPs were synthesized via the solvothermal method, followed by replacing the alcohol dispersion medium with water [27]. During this process, Ta–O–Ta bonds are formed through the reaction of two tantalum ethoxide molecules via ether elimination. Among various synthesis methods for producing tantalum oxide NPs, this approach offers a significant advantage by enabling the formation of stable aqueous dispersions of nanoscale particles without requiring stabilizers. This allowed us to study the properties of the bare surface, since the absence of stabilizers preserved the intrinsic characteristics of the material, providing valuable insights into its behavior. This was especially essential for conducting a rigorous comparative analysis between coated and uncoated nanomaterial samples.

**Figure 1:**
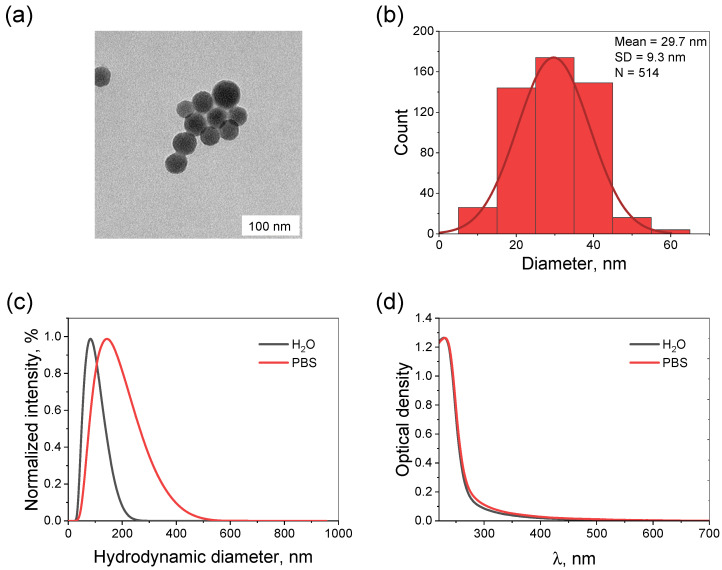
Characterization of the synthesized Ta_2_O_5_ NPs. (**a**) TEM image and (**b**) corresponding size distribution histogram (514 particles were analyzed). (**c**) Size distributions measured by DLS and (**d**) extinction spectra of Ta_2_O_5_ NPs (1.5 × 10^−9^ M). The color legends in figures (**c**,**d**) are identical: gray curves correspond to distilled water, and those of red—to PBS.

To visualize the synthesized NPs, a transmission electron microscopy (TEM) analysis was performed (Figure 1a). The uncoated Ta_2_O_5_ NPs exhibited a spherical morphology with an average core size of 29.7 ± 9.3 nm (the corresponding size distribution is shown in Figure 1b). The hydrodynamic diameter of the NPs was measured in distilled water and phosphate-buffered saline (PBS) using dynamic light scattering (DLS; Figure 1c). In water, it was found to be 70.4 ± 0.3 nm with a corresponding polydispersity index (PDI) of 0.235. In this case, the NPs were characterized by a zeta potential value of −49.3 ± 1.5 mV. This parameter is one of the key features indicating the colloidal stability of dispersed systems. For these NPs, as well as for their various modifications described below, all results on hydrodynamic diameters and PDI are summarized in Table S1. In turn, in PBS, the hydrodynamic diameter of the NPs increased to 129.1 ± 0.7 nm, with the PDI increasing to 0.191. Their electrical double layer was compressed, resulting in a decrease in the zeta potential to −30.4 ± 1.3 mV. All this clearly indicated the aggregation of the uncoated Ta_2_O_5_ NPs. However, no noticeable differences were observed between the studied solutions by the extinction spectra (Figure 1d).

According to the literature, DA and PDA attach to the surface of metal oxides NPs through the complexation reaction, which involves the replacement of surface oxide hydroxyl groups by deprotonated catechol groups from the ligand [28,29]. Thus, to coat the NPs with a PDA layer, a dopamine (DA) solution was mixed with Ta_2_O_5_ hydrosol. Then, the pH of the mixture was then adjusted to approximately 8.5 to initiate DA polymerization. The results of the study of the tantalum oxide NPs before and after their coating with PDA via Fourier transform infrared spectroscopy (FTIR) are presented in the Supplementary Materials (Figure S1). A series of new peaks which appeared in the range of 1250–1550 cm^−1^ clearly indicated the adsorption of PDA on the NP surface [30]. The formation of the PDA coating was further confirmed by XPS analysis, which revealed nitrogen-containing functional groups characteristic of polydopamine (Figure S2).

**Figure 2:**
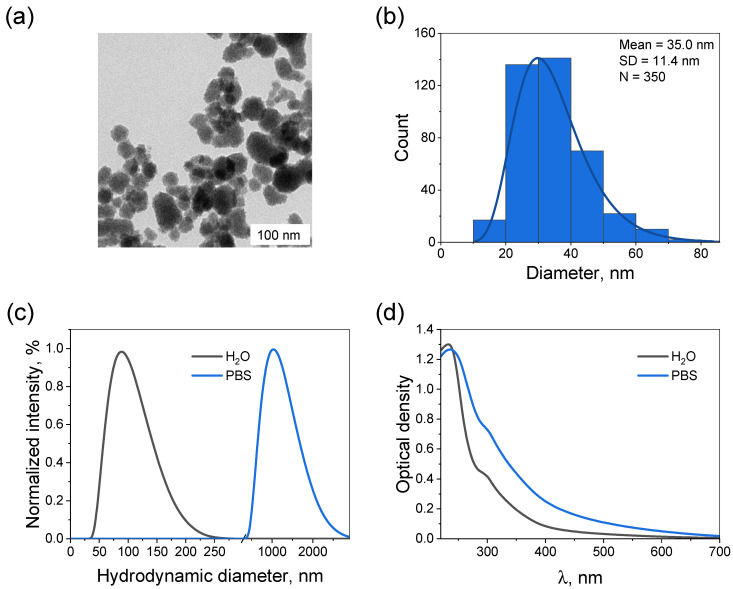
Characterization of the obtained Ta_2_O_5_@PDA NPs. (**a**) TEM image and (**b**) corresponding size distribution histogram (350 particles were analyzed). (**c**) Size distributions measured by DLS and (**d**) extinction spectra of Ta_2_O_5_ NPs (1.1 × 10^−9^ M). The color legends in figures (**c**,**d**) are identical: gray curves correspond to distilled water, and those of blue—to PBS.

The corresponding TEM image for the PDA-coated NPs is presented in Figure 2a. It can be seen that after the deposition of PDA, the NP surface became quite rough. According to the statistical analysis, the average size of the NPs increased to 35.0 ± 11.4 nm after their modification (Figure 2b). After coating, the hydrodynamic diameter measured in water also increased to 86.5 ± 0.4 nm (Figure 2c). In turn, the values of PDI and zeta potential reached 0.104 and −46.9 ± 1.8 mV, respectively. Thus, coating the NPs with PDA did not lead to a decrease in their stability in distilled water. However, a significantly different picture was observed in PBS. Here, the PDA-modified NPs exhibited a drastic growth to 911.8 ± 44.0 nm. Such sharp increase in the average particle size, as well as the broadening of the size distribution clearly indicated the strong aggregation of the NPs. This finding was further supported by the alterations in the extinction spectrum upon changing the solvent from distilled water to PBS (Figure 2d). The observed increase in the signal, especially in the visible region of the spectrum, where there is no absorption by the NPs themselves, may be associated with an increase in the contribution from light scattering by their large aggregates. In turn, the shoulder observed near 300 nm corresponded to the 5,6-dihydroxyindole units in the PDA molecules [31]. Herewith, the zeta potential of the Ta_2_O_5_@PDA NPs in PBS was −30.9 ± 0.3 mV, indicating that electrostatic interactions are not the only factor affecting their stability. In general, such behavior was consistent with the report for the PDA-coated gold NPs [32]: they also suffered a salt-induced aggregation, the effect of which was enhanced by the PDA layer.

Interaction of BSA with the uncoated and coated tantalum oxide NPs was investigated by measuring the quenching of its fluorescence. It is well-known that photophysical properties of serum albumins are determined by their composition, namely, by the presence of such amino acids as tryptophan (Trp), tyrosine (Tyr) and phenylalanine (Phe) [33]. The main contribution to the signal comes from Trp residues. In native proteins, Tyr emission is often suppressed, presumably by its interaction with the peptide chain or via energy transfer to Trp. In turn, Phe has a low quantum yield and thus contributes minimally. Upon excitation at 280 nm, both Trp and Tyr residues fluoresce, while irradiation at 295 nm selectively excites Trp. Thus, data obtained at both wavelengths may provide complementary information about the microenvironment of these residues [34]. Based on this rationale, for both NP types, the efficacy of BSA fluorescence quenching was investigated at excitation wavelengths of 280 and 295 nm.

In order to eliminate the inner-filter effects, the fluorescence intensities were corrected by the formula below [35,36]:

(1) $$F_{corr}=F_{obs}\times 10^{\frac{A_{ex}}{2}+\frac{A_{em}}{2}}\;$$

where $F_{corr}$ and $F_{obs}$ are the corrected and observed fluorescence intensities, $A_{ex}$ and $A_{em}$ are the absorption intensities at the given excitation and emission wavelengths, respectively.

The quenching data were analyzed by the Stern–Volmer equation [35]:

(2) $$\frac{F_{0}}{F}=1+K_{SV}\;\left[Q\right]$$

where $F_{0}$ and F are the fluorescence intensities of BSA in the absence and presence of the NPs, $K_{SV}$ is the Stern−Volmer constant, and [Q] is the NP concentration.

The quenching constant $k_{q}$ was determined as follows:

(3) $$k_{q}=\frac{K_{SV}}{\tau_{0}},$$

where $\tau_{0}$ is the average excited state lifetime of BSA (6.19 ns), measured in the absence of NPs.

**Figure 3:**
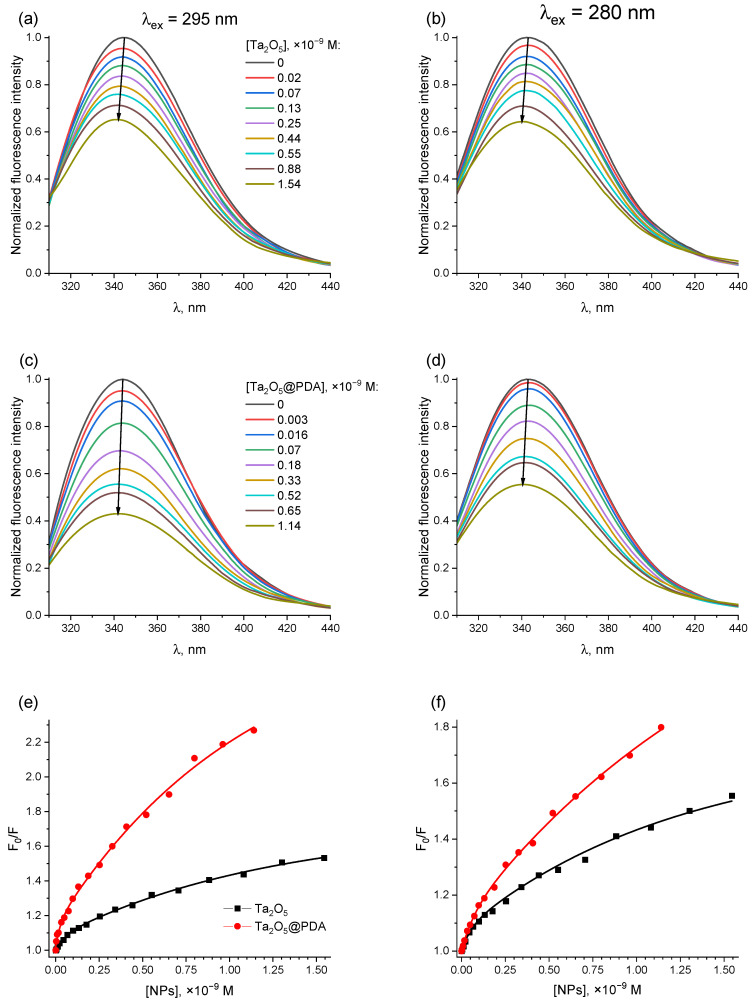
(**a**–**d**) Normalized fluorescence spectra of BSA (2 × 10^−6^ M) in distilled water in the presence of (0–1.53) × 10^−9^ M of Ta_2_O_5_ NPs (**a**,**b**) and (0–1.14) × 10^−9^ M of Ta_2_O_5_@PDA NPs (**c**,**d**). (**e**,**f**) Stern–Volmer plots for the quenching of BSA fluorescence by Ta_2_O_5_ (black curves) and Ta_2_O_5_@PDA (red curves) NPs. The left column shows the data for the case of the protein excitation at 295 nm, and the right one—for 280 nm, respectively.

Figure 3a–d present the normalized fluorescence spectra of aqueous solutions of BSA (2 × 10^−6^ M) in the presence of increasing concentrations of Ta_2_O_5_ or Ta_2_O_5_@PDA NPs (up to 1.54 × 10^−9^ M and 1.14 × 10^−9^ M, respectively). It can be seen that both when the protein fluorescence was excited at a wavelength of 280 nm, and when it was excited at 295 nm, the signal intensity decreased with an increase in the content of NPs in the system. In both cases, a significantly stronger drop was observed for Ta_2_O_5_@PDA NPs. Thus, upon excitation at 295 nm, the fluorescence intensity decreased by 1.5 times at the bare NP concentration of 1.32 × 10^−9^ M, whereas for PDA-coated ones, a similar effect was observed at 0.25 × 10^−9^ M (upon excitation at 280 nm, the corresponding concentrations were 1.33 × 10^−9^ M and 0.57 × 10^−9^ M, respectively). Plotting the $\frac{F_{0}}{F}$ ratio against the NP concentration provided a much clearer comparison (Figure 3e,f). The corresponding values of Stern–Volmer constant, estimated from linear sections of these dependencies, are summarized in Table 1. The values of quenching constant are also given there. Note that for both types of NPs, a slight shift in the spectral maximum to the blue region was noted. Among others, this could be due to changes in the microenvironment of the fluorophores due to the particle binding [34].

For the case of fluorescence excitation at 295 nm, the $K_{SV}$ value for the PDA-coated NPs was approximately 2.5-fold higher than that for the uncoated particles. A less pronounced difference was found when it was excited at a wavelength of 280 nm. This variation may indicate a stronger affinity of Ta_2_O_5_@PDA NPs for BSA compared to bare Ta_2_O_5_ NPs. In turn, the $k_{q}$ values for both types of the NPs significantly exceeded the diffusion-controlled limit (1–2) × 10^10^ M^−1^ s^−1^ [37,38], indicating that the observed quenching of BSA fluorescence might have not only collisional nature, but also involve binding interactions [35].

The Stern–Volmer and quenching constants for Ta_2_O_5_ (*K*_SV_ = 1.25 × 10^9^ M^−1^) and Ta_2_O_5_@PDA (*K*_SV_ = 3.12 × 10^9^ M^−1^) NPs exceed those reported for titanium dioxide (*K*_SV_ ≈ 0.5 × 10^4^ M^−1^) [39] and iron oxide (*K*_SV_ ≈ 10^4^–10^8^ M^−1^) [40,41,42] NPs, which are among the most extensively studied metal oxide systems. At the same time, these values are lower than or comparable to those of plasmonic NPs such as gold and silver (*K*_SV_ ≈ 10^7^–10^10^ M^−1^) [43], which exhibit enhanced quenching efficiency due to localized surface plasmon resonance effects. Comparative studies on uncoated and PDA-coated iron oxide NPs [34] and functionalized metal–polyphenol NPs [44] reported differences in quenching efficacy related to surface modification. Thus, a slight decrease in the effect was observed for PDA-coated NPs in these studies. This result may be due to a reduction in the surface area of NPs available for adsorption, which in turn could be a result of their aggregation in buffer solutions [44]. Additionally, the coating formation may stimulate small particles to cluster into larger aggregates [34]. Such aggregation reduces the total surface area accessible for protein binding, thereby lowering the fluorescence quenching efficiency. However, as we noted above, the efficacy of fluorescence quenching can be affected by various factors (e.g., particle size, crystallinity, surface charge, etc. For more detail, see [25]); therefore, even within the same class of NPs, the values of the corresponding constants may differ by many orders of magnitude.

Let us now consider the quenching of BSA fluorescence in more detail. For both studied types of NPs, the signal dropped rapidly at low concentrations and decreased noticeably more slowly at higher concentrations, resulting in a downward-curving Stern−Volmer plot. The observed negative deviation from linearity suggested heterogeneity in the fluorophore population, where only a fraction of fluorophores contributes significantly to the fluorescence signal [45]. To estimate the fraction of fluorophores accessible to quenching, the modified Stern−Volmer equation can be used [35,46]:

(4) $$\frac{F_{0}}{F_{0}-F}=\frac{1}{f_{a}}+\frac{1}{f_{a}K_{q}}\frac{1}{\left[Q\right]},$$

where $f_{a}$ represents the fraction of fluorophore accessible to the NPs and $K_{q}$ is the apparent quenching constant; $F_{0}$, F and $\left[Q\right]$ are the same as in Equation (2).

The analysis using Equation (4) did not provide us with convincing data, as the results were heavily distorted by errors. This is most likely due to the fact that Equation (4) is a linear transformation of the equation describing the quenching of heterogeneously emitting systems [47]:

(5) $$\frac{F}{F_{0}}=\sum_{i=1}^{n}\frac{f_{i}}{1+K_{q,i}\left[Q\right]},$$

This is consistent with the fact that linear transformations can sometimes distort data and lead to unsatisfactory results [48,49]. Therefore, the use of non-linear approximation methods may be more appropriate in some cases [49]. For analysis, we used Equation (6) [50], which is a mathematically equivalent non-linear transformation of Equation (4). This equation is also a special case of Equation (5), assuming the presence of only two types of fluorophore populations:

(6) $$\frac{F}{F_{0}}=\left(1-f_{a}\right)+\frac{f_{a}}{1+K_{q}\left[Q\right]},$$

**Figure 4:**
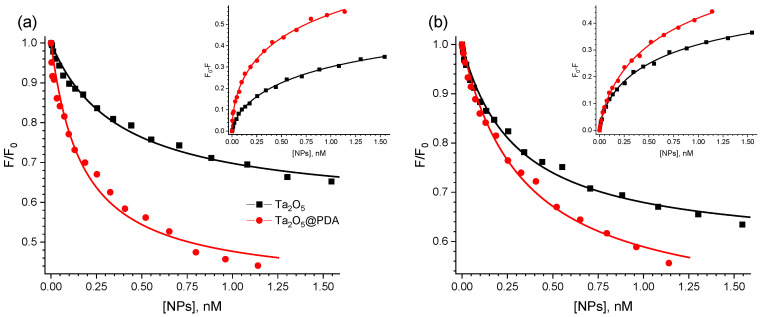
Modified Stern−Volmer plots for the quenching of BSA fluorescence by Ta_2_O_5_ (black curves) and Ta_2_O_5_@PDA (red curves) NPs. Panel (**a**) shows the data for the case of the protein excitation at 295 nm, and panel (**b**)—for 280 nm, respectively. The insets show the corresponding Hill plots for the quenching by Ta_2_O_5_ (black curves) and Ta_2_O_5_@PDA (red curves) NPs at 295 nm (**a**) and 280 nm (**b**).

The plots of $\frac{F}{F_{0}}$ as a function of $\left[Q\right]$, constructed based on the mathematical model represented by Equation (6), are shown in Figure 4. The respective quantitative parameters $K_{q}$ and $f_{a}$ calculated according to this model are summarized in Table 2.

The values of constant calculated according to Equation (6) were higher compared to those calculated by Equation (2). Modification of the NPs with PDA led to a slight increase of $f_{a}$: from 0.42 to 0.55 and from 0.45 to 0.61 upon excitation of the protein fluorescence at wavelengths of 280 and 295 nm, respectively. Thus, it did not drastically influence the accessibility of fluorophores in BSA molecules.

Continuing the discussion regarding the mechanisms of protein–NP interaction, we suggest that the Hill equation could provide valuable additional information about these mechanisms:

(7) $$log\left(\frac{F_{0}-F}{F}\right)=logK_{b}+nlog\left[Q\right],$$

In the literature, this is sometimes termed the double log Stern–Volmer equation [51]. However, it is fundamentally a linear transformation of the original Hill equation [52]. The conventional form of Equation (7) describes the limiting case of a completely quenched complex. Since we observed incomplete fluorophore accessibility, the equation must be corrected for the residual fluorescence intensity at saturation (F_∞_) [53]. Additionally, we directly used the non-linear form of the modified Hill equation, as the linear regression of transformed data is not the optimal method for parameter estimation [54]. Despite the widespread use of the linear transformation form, the application of non-linear fitting to fluorescence quenching data, particularly for NP systems, remains relatively uncommon [55]. The adapted non-linear equation is given as

(8) F0−FF0−F∞=Qn1Kbn+Qn,

where n is the Hill coefficient, $K_{b}\;$is the overall binding constant, F_∞_—intensity of residual fluorescence; $F_{0}$, F and $\left[Q\right]$ are the same as in Equations (2) and (6). The corresponding plots are shown in Figure 4 (inset), and the calculated values of n and $K_{b}$ are presented in Table 3.

Although many studies interpret the coefficient *n* as the number of binding sites [51,56], there is substantial evidence that *n* is not a stoichiometric coefficient but a phenomenological parameter (the Hill coefficient), which is often lower than the actual number of binding sites [52,54,57]. The Hill coefficient serves as a model-independent measure of cooperativity within a system under equilibrium conditions and quantifies cooperative interactions between binding sites [58]. The value of *n* < 1 indicates negative cooperativity, where the binding of one ligand molecule impedes the binding of another one. For instance, in the context of protein adsorption onto NPs, this manifests as a gradual decrease in the binding affinity for additional protein molecules on the same particle surface. Conversely, *n* > 1 indicates positive cooperativity, meaning the adsorption of one protein molecule enhances the attachment of others to the same NP surface. Interestingly, the Hill coefficients *n* in our systems were found to be significantly below unity, suggesting negative cooperativity in the binding process. This observation is consistent with the downward-curving shape of the Stern–Volmer plots (Figure 3e,f). The more pronounced difference in quenching constants observed at 295 nm excitation compared to 280 nm across all three analytical approaches (Stern–Volmer, modified Stern–Volmer, and Hill equations), likely indicates a stronger interaction between the coated NPs and the tryptophan residues of the protein.

**Figure 5:**
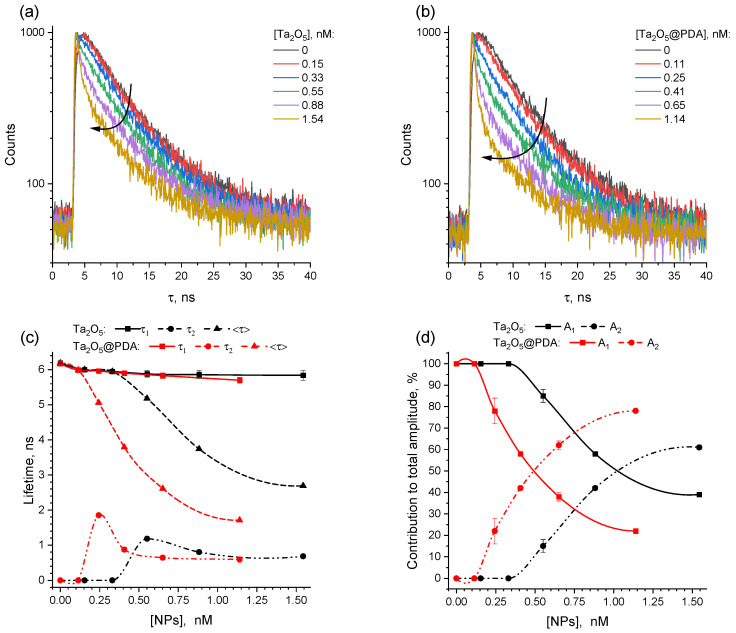
(**a**,**b**) Fluorescence decay kinetics of BSA (λ_ex_ = 300 nm, λ_em_ = 345 nm) in distilled water in the presence of (0–1.53) × 10^−9^ M of Ta_2_O_5_ (**a**) and (0–1.14) × 10^−9^ M of Ta_2_O_5_@PDA (**b**) NPs. The color legend in these figures is identical. The arrow indicates the increase of concentration of NPs. (**c**,**d**) Dependences of the lifetimes ((**c**); τ_1_—squares, τ_2_—circles, <τ>—triangles) and their contributions to the total fluorescence amplitude ((**d**); A_1_—squares, A_2_—circles) on the concentration of Ta_2_O_5_ NPs (black curves) and Ta_2_O_5_@PDA NPs (red curves).

Figure 5 represents the fluorescence decay kinetics of BSA in distilled water in the presence of various concentrations of Ta_2_O_5_ NPs and Ta_2_O_5_@PDA NPs. The curves were analyzed using the two-exponential model, with the exception of native protein samples, which were analyzed using a single-exponential model:

(9) It=A1e−tτ1+A1e−tτ2,

where $\tau_{1}$ and $\tau_{2}$ are the lifetime components, $A_{1}$ and $A_{1}$ are their contributions to the total fluorescence amplitude.

The average fluorescence lifetime <τ> was calculated from the following equation:

(10) $$<\tau >=\frac{\sum_{i}A_{i\;}\tau_{i}^{2}}{\sum_{i}A_{i\;}\tau_{i}}$$

The variations of $\tau_{1}$ and $\tau_{2}$, as well as $A_{1}$ and $A_{1}$, are shown in Figure 5c,d. In the absence of NPs, the decay curve for BSA in water is monoexponential, with a single lifetime *τ* ≈ 6.19 ns, which is in good agreement with the literature [59,60]. A similar shape of the curves was also observed at low concentration of the NPs. However, in these cases, the lifetime gradually decreased from 6.19 ns to 5.95 ns (bare Ta_2_O_5_) and from 6.17 ns to 5.99 ns (Ta_2_O_5_@PDA). In turn, to accurately fit the experimental curves at increasing NP concentration, a two-component exponential model was required. Herewith, the short-lived component can be attributed to bound BSA molecules, thereby supporting the existence of binding interactions. Indeed, as the NP concentration increases, its contribution to the total fluorescence amplitude increased, while that of the long-lived one gradually decreased. Moreover, the average fluorescence lifetime <τ> demonstrated a noticeable reduction with rising NP concentration, particularly for coated particles.

It is also well-known that a change in the lifetime is the characteristic feature of Förster resonance energy transfer (FRET). Since FRET occurs at very close distances (≤10 nm), it may also be a marker of complex formation between the NPs and BSA. For the PDA-coated NPs, the corresponding changes were more pronounced, which may indicate more efficient energy transfer (note that the contribution of the short-lived component to the total intensity is also greater for Ta_2_O_5_@PDA).

**Figure 6:**
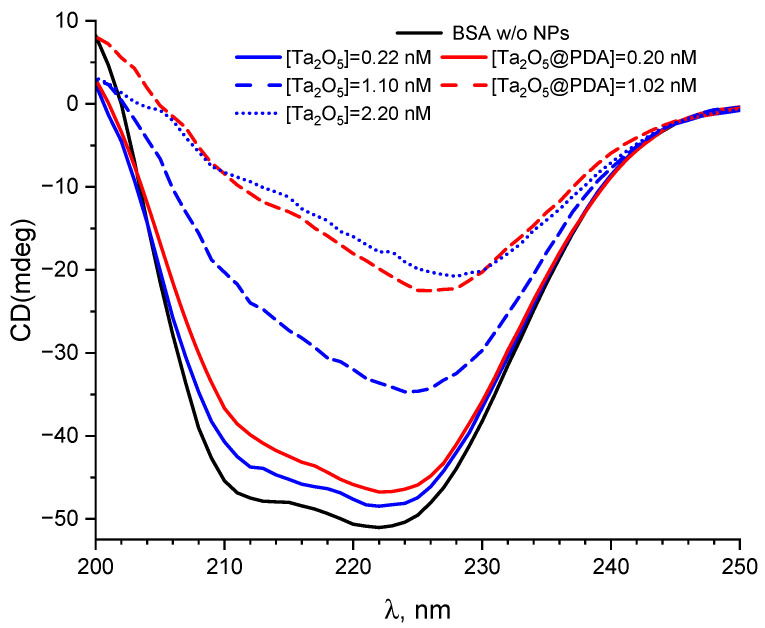
CD spectra of 2 × 10^−6^ M BSA in the absence (black line, BSA w/o NPs) and presence of varying concentrations of bare Ta_2_O_5_ NPs (blue lines) and Ta_2_O_5_@PDA NPs (red lines).

Further experiments were carried out using circular dichroism (CD) spectroscopy, which is an excellent method for analyzing the structure (conformation) of proteins and peptides in a solution [61]. The CD spectra recorded for 4.5 × 10^−6^ M BSA in the absence and presence of varying concentrations of Ta_2_O_5_ and Ta_2_O_5_@PDA NPs are shown in Figure 6. Native BSA exhibited characteristic CD minima at 211.5 nm and 222 nm (black curve). The slight shift in the shorter-wavelength minimum from the typically reported 208 nm (in buffered solutions) to 211.5 nm is attributed to the usage of water as a solvent. Upon addition of NPs, the intensity of both minima decreased, indicating a loss of α-helical content and suggesting structural changes [62] consistent with NP-BSA complex formation [63]. The α-helix content in native BSA was 54% (it was calculated at wavelength 222 nm, according to [64]), which is consistent with literature values [65,66]. CD spectroscopy revealed partial unfolding of BSA upon interaction with NPs, characterized by a substantial loss of α-helical content and CD-signal intensity at 222 nm [67,68]. The effect was concentration-dependent and significantly more pronounced for PDA-coated NPs. At 1 nM, Ta_2_O_5_@PDA induced a ≈40% reduction in helicity and a red shift in the longer-wavelength minimum to ≈227 nm, whereas bare Ta_2_O_5_ caused a similar change only at 2 nM. This indicates that the PDA coating may enhance the unfolding of BSA by the NPs. The decrease in the 208/220 nm ellipticity ratio from 0.83 (native) to 0.55 (Ta_2_O_5_) and 0.36 (Ta_2_O_5_@PDA) further confirms the loss of ordered structure [69]. In contrast, no such structural changes were detected for iron oxide nanoparticles, either coated with PDA or uncoated [34].

**Figure 7:**
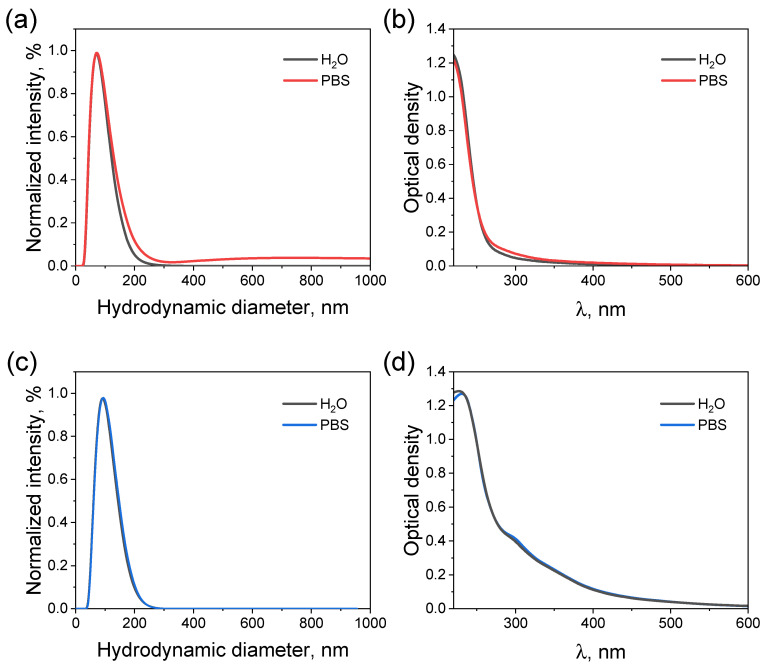
Characterization of the synthesized BSA-coated Ta_2_O_5_ and Ta_2_O_5_@PDA NPs. (**a**,**c**) Size distributions measured by DLS and (**b**,**d**) extinction spectra of the NPs (≈1.5 × 10^−9^ M for Ta_2_O_5_ and ≈1.1 × 10^−9^ M for Ta_2_O_5_@PDA NPs). The top row shows the data for Ta_2_O_5_ NPs coated with BSA, and the one—for Ta_2_O_5_@PDA-BSA NPs. The black curves correspond to distilled water, and those of red and blue—to PBS.

Then, knowing that BSA successfully binds to studied NPs, let us assess their colloidal stability. After modification, the hydrodynamic diameter of Ta_2_O_5_ and Ta_2_O_5_@PDA NPs in distilled water increased to 72.4 ± 0.7 nm and to 89.1 ± 0.4 nm, respectively. The corresponding PDI values were 0.190 and 0.101. The zeta potential values for Ta_2_O_5_-BSA and Ta_2_O_5_@PDA-BSA NPs remained highly negative (−40.3 ± 2.4 and −47.3 ± 1.1 mV), which favored electrostatic stabilization. The formation of the adsorbed BSA layer was also supported by FTIR spectroscopy, as evidenced by the intensified absorption band at 1648 cm^−1^, characteristic of the C=O stretching vibrations in the amide I region (Figure S1) [70]. The colloidal stability of Ta_2_O_5_-BSA and Ta_2_O_5_@PDA-BSA NPs was studied in PBS (Figure 7). Overall, it can be seen that being coated with BSA significantly improved their stability in this medium. However, there were some differences in their behavior. While the size distributions of the double-modified NPs in PBS closely matched those measured in distilled water (Figure 7c), Ta_2_O_5_-BSA NPs showed signs of slight aggregation, as evidenced by a minor broadening of the size distribution peak and the appearance of a very broad band at higher diameters (Figure 7a). The zeta potentials values of Ta_2_O_5_-BSA and Ta_2_O_5_@PDA-BSA NPs in PBS were found to be −39.0 ± 3.5 and −25.2 ± 2.1 mV, respectively. Considering that the zeta potential of the latter was relatively low, it can be assumed that a steric mechanism makes a significant contribution to the stability of the system. In turn, no noticeable difference in the absorption spectra of the BSA-modified particles was observed between water and PBS (Figure 7b,d).

## 3. Materials and Methods

### 3.1. Materials

Tantalum (V) ethoxide (99.98%; Sigma-Aldrich, St. Louis, MO, USA), isopropyl alcohol (IPA; Chimmed, Moscow, Russia), sodium hydroxide solution (1 M) and DA hydrochloride (ACS Reagent; Sigma-Aldrich, USA), ready-made PBS with pH of 7.2–7.6 (Eco Service, Moscow, Russia), and BSA (PanEco, Moscow, Russia) were used as received. The protein concentration was determined spectrophotometrically using the known extinction coefficient ε_280_ = 44,720 M^−1^ cm^−1^ [71].

### 3.2. Synthesis of Ta_2_O_5_ NPs

Ta_2_O_5_ NPs were synthesized via the solvothermal method according to the protocol described in detail by Koshevaya et al. [27]. Briefly, 0.468 mL of tantalum (V) ethoxide was added in a drop-wise manner to 45 mL of anhydrous IPA. All manipulations with Ta(OC_2_H_5_)_5_ were performed under a nitrogen atmosphere to prevent uncontrolled hydrolysis reaction. Next, a Teflon cup filled with the resulting solution was transferred to a stainless-steel autoclave and carefully sealed. The autoclave was placed in a furnace and heated to 200 °C for 12 h. This resulted in a transparent sol of primary particles in IPA. To obtain a hydrosol of Ta_2_O_5_ NPs, the sol of primary particles in IPA was mixed with water deionized using Arium 611 device (Sartorius, Göttingen, Germany) in such a volume ratio as to ultimately achieve a tantalum oxide content of 10 mg/mL (1.5 × 10^−7^ M of Ta_2_O_5_ NPs). Then, the obtained mixture was slowly heated to remove IPA. During evaporation, the temperature was kept below the boiling point of IPA (82.5 °C).

### 3.3. Surface Modification of Ta_2_O_5_ NPs with PDA

To prepare Ta_2_O_5_@PDA NPs, the Ta_2_O_5_ hydrosol (3 mg/mL or 4.4 × 10^−8^ M) and DA solution (0.5 mg/mL) were mixed at a volume ratio of 1:1. To start the polymerization of DA, NaOH solution of 0.1 M was added to the dispersion in a drop-wise manner until its pH reached ≈8.5. Then, the mixture was placed in a Rotamix shaker (Elmi, Riga, Latvia) and slowly rotated (20 rpm) at room temperature for 3 h. The prepared PDA-coated NPs were centrifuged at 14,000 rpm for 20 min using 320R centrifuge (Hettich, Tuttlingen, Germany). After that, the precipitated particles were resuspended in deionized water (30 s of ultrasonication using a Elmasonic S10H device (Elma, Singen, Germany)). This cycle was repeated 3 times.

### 3.4. Determination of NP Molar Concentration

The molar concentration of Ta_2_O_5_ and Ta_2_O_5_@PDA NPs was calculated based on the particle number. Individual particle diameters were obtained from TEM, yielding mean diameters of ⟨d⟩ = 29.7 nm and ⟨d⟩ = 35.0 nm, respectively. Assuming spherical geometry, the average particle volume was calculated as

V = (π/6) × ⟨d⟩^3^

Particle mass for Ta_2_O_5_ NPs was determined as m(NP) = ρ × V, where ρ is the density of Ta_2_O_5_ (8.2 g/cm^3^) and V is the average particle volume.

Particle mass m(NP) for Ta_2_O_5_@PDA NPS was determined as the sum of the oxide core mass and PDA-layer mass. The PDA-layer mass is determined as m(PDA) = ρ × V, where ρ is the density of PDA-layer (1 g/cm^3^) and V is the average layer volume. The PDA-layer volume was calculated as the difference between the volumes of spheres with diameter of 35.0 nm (oxide core + PDA shell) and 29.7 nm (oxide core), corresponding to a shell thickness of approximately 2.7 nm.

The particle number in a given solution volume was determined from

N = m(total)/m(NP)

where m(total) is the mass of Ta_2_O_5_ and Ta_2_O_5_@PDA determined gravimetrically. The molar concentration was finally calculated as c = N/N_A_, where N_A_ = 6.022 × 10^23^ mol^−1^ is Avogadro’s number.

This calculation did not consider the polydispersity of the particle size distribution, which is a limitation of the approach used.

### 3.5. Surface Modification of Ta_2_O_5_ and Ta_2_O_5_@PDA NPs with BSA

Ta_2_O_5_-BSA and Ta_2_O_5_@PDA-BSA NPs were prepared by mixing hydrosols of NPs, aqueous solutions of BSA and NaOH at the following concentrations: 0.75 mg/mL of NPs (1.1 × 10^−8^ M of Ta_2_O_5_ NPs and 1.0 × 10^−8^ M of Ta_2_O_5_@PDA NPs), 3.3 mg/mL of BSA (50 μM), 3 mM of NaOH. The resulting mixtures were characterized by a pH of ≈8.5. Then, they were placed in the shaker and slowly rotated at room temperature for 4 h using the same settings as above. Finally, the NPs were centrifuged and washed three times with deionized water to remove unbound BSA and impurities.

### 3.6. Characterization of NPs

#### 3.6.1. Transmission Electron Microscopy

The size and morphology of Ta_2_O_5_ and Ta_2_O_5_@PDA NPs were determined using a high-resolution transmission electron microscope (HRTEM) Libra 120 (C. Zeiss, Oberkochen, Germany) operating at an accelerating voltage of 120 kV. For this purpose, a drop of the corresponding dispersion was placed on a formvar-coated copper grid for 1 min and then removed with filter paper. Particle size distribution histograms were obtained by counting at least 350 particles. The particle size was analyzed using an ImageJ.JS software (National Institutes of Health, Bethesda, MD, USA).

#### 3.6.2. FTIR Spectroscopy

The formation of PDA and BSA layers on the NPs was qualitatively evaluated using Nicolet 380 FTIR spectrometer (Thermo Electron Corporation, Waltham, MA, USA). The spectra were measured in the diffuse reflectance mode in the wavenumber range of 400–4000 cm^−1^.

#### 3.6.3. Dynamic Light Scattering and Laser Doppler Electrophoresis

The hydrodynamic diameters of the synthetized NPs, as well as their zeta potentials in various solvents, were determined via Zetasizer Nano ZS spectrometer (Malvern, Malvern, UK) with solid state He–Ne laser generating light at a wavelength of 633 nm (scattering angle of 173°). In all cases, the concentration of NPs in the samples was (1.5–3.0) × 10^−8^ M of Ta_2_O_5_ NPs and (1.1–2.2) × 10^−8^ M of Ta_2_O_5_@PDA NPs). The measurements were carried out in the special quartz cells at 25 °C. The presented results were averaged over five independent measurements.

#### 3.6.4. Absorbance, Fluorescence and Circular Dichroism Measurements

Absorption and steady-state fluorescence spectra were recorded using a UV-3101 PC spectrophotometer and an RF5301PC spectrofluorimeter (Shimadzu, Kyoto, Japan). In this case, BSA fluorescence was excited at wavelengths of 280 and 295 nm. Time-resolved fluorescence measurements were performed by the time-correlated single photon-counting (TCSPC) method using a FluoTime 300 spectrometer (Picoquant, Berlin, Germany; a pulsed 300 nm laser light source was used for the protein excitation, and its fluorescence was detected at a wavelength of 345 nm). The decay curves were fitted by the exponential model using EasyTau 2 software. When calculating the excited state lifetimes, *χ*^2^ did not exceed 1.3. In all cases, the measurements were performed in rectangular quartz cells (Hellma, Müllheim, Germany) with 1 cm path length for excitation light. Circular dichroism (CD) measurements were carried out on a J-715 spectropolarimeter (Jasco, Tokyo, Japan) at 20 °C using a quartz cell with 0.1 cm path length (Hellma, Müllheim, Germany).

## 4. Conclusions

This study provides the first comprehensive investigation of the interactions between tantalum oxide NPs and BSA. Using spectroscopic methods, we demonstrated that both bare and PDA-coated Ta_2_O_5_ NPs bind BSA quite effectively. Our findings revealed that the PDA coating acts as a crucial functional layer, significantly enhancing the binding affinity to BSA, as evidenced, among other things, by the increase in Stern–Volmer constant. The existence of FRET between BSA molecules and NPs also confirmed our assumptions. Analysis via Hill equation showed a negative binding cooperativity (*n* < 1) for both Ta_2_O_5_ and Ta_2_O_5_@PDA NPs, suggesting steric hindrance as a limiting factor for fluorophore accessibility. CD data demonstrated a significant disruption of BSA’s secondary structure upon NP binding, consistent with partial unfolding. The PDA-coated NPs (Ta_2_O_5_@PDA) induced a markedly greater loss of α-helical content and a more drastic change in the 208/220 nm ellipticity ratio compared to bare Ta_2_O_5_ NPs. This indicates that the PDA coating may enhance the unfolding of BSA by the NPs.

Moreover, it was shown that surface modification with BSA significantly enhances the colloidal stability of the studied NPs in high-ionic strength environments. While bare Ta_2_O_5_ and Ta_2_O_5_@PDA NPs exhibited considerable aggregation in PBS, those coated with BSA demonstrated markedly reduced aggregation or its complete absence. The enhanced stability may be attributed to the synergism of electrostatic and steric stabilization mechanisms provided by the adsorbed BSA layer.

In conclusion, this work fills a critical knowledge gap in understanding the interaction between Ta_2_O_5_ NPs and albumins and establishes an effective strategy for NP stabilization in high ionic strength environments via sequential PDA and BSA coatings. The functional groups present in both PDA and BSA offer versatile platforms for further modification, enabling the fabrication of multifunctional nanostructures suitable for advanced biomedical applications such as targeted imaging, therapeutic delivery, and stimuli-responsive systems [72,73,74,75].

## Figures and Tables

**Table 1:** Calculated values of Stern−Volmer and quenching constants for the Ta_2_O_5_ and Ta_2_O_5_@PDA NPs.

NPs	$K_{SV}$, × 10^9^, (M^−1^)		$k_{q}$, × 10^17^, (M^−1^ s^−1^)	
	$\lambda_{ex}$ = 280 nm	$\lambda_{ex}$ = 295 nm	$\lambda_{ex}$ = 280 nm	$\lambda_{ex}$ = 295 nm
Ta_2_O_5_	1.17 ± 0.04	1.25 ± 0.06	1.89 ± 0.06	2.02 ± 0.10
Ta_2_O_5_@PDA	1.93 ± 0.11	3.12 ± 0.23	3.12 ± 0.18	5.04 ± 0.37

**Table 2:** Calculated values of apparent quenching constants and accessible fraction of the fluorophore for the Ta_2_O_5_ and Ta_2_O_5_@PDA NPs.

NPs	$\lambda_{ex}$ = 280 nm		$\lambda_{ex}$ = 295 nm	
	$K_{q}$, × 10^9^, (M^−1^)	$f_{a}$	$K_{q}$, × 10^9^, (M^−1^)	$f_{a}$
Ta_2_O_5_	2.39 ± 0.19	0.42 ± 0.01	2.63 ± 0.24	0.45 ± 0.02
Ta_2_O_5_@PDA	3.32 ± 0.29	0.55 ± 0.02	4.94 ± 0.69	0.61 ± 0.03

**Table 3:** Calculated values of Hill coefficient and binding constant for Ta_2_O_5_ and Ta_2_O_5_@PDA NPs.

NPs	$\lambda_{ex}$ = 280 nm		$\lambda_{ex}$ = 295 nm	
	$K_{b}$, × 10^9^, (M^−1^)	n	$K_{b}$, × 10^9^, (M^−1^)	n
Ta_2_O_5_	0.44 ± 0.12	0.65 ± 0.02	0.46 ± 0.10	0.68 ± 0.03
Ta_2_O_5_@PDA	0.63 ± 0.15	0.67 ± 0.03	1.33 ± 0.03	0.59 ± 0.02

## Data Availability

The original contributions presented in this study are included in the article/Supplementary Materials. Further inquiries can be directed to the corresponding author.

## Supplementary Materials

The following supporting information can be downloaded at: https://www.mdpi.com/article/10.3390/molecules31020241/s1, Table S1: Hydrodynamic diameters and polydispersity index of NPs in water and PBS; Figure S1: FTIR spectra of Ta_2_O_5_, Ta_2_O_5_@PDA, Ta_2_O_5_-BSA and Ta_2_O_5_@PDA-BSA NPs; Figure S2: XPS spectra in the N1s region for bare Ta_2_O_5_ NPs and Ta_2_O_5_@PDA NPs.

## References

[1] Shan X., Gong X., Li J., Wen J., Li Y., Zhang Z. (2022). Current Approaches of Nanomedicines in the Market and Various Stage of Clinical Translation. Acta Pharm. Sin. B.

[2] Mashtalyar D.V., Imshinetskiy I.M., Kashepa V.V., Nadaraia K.V., Piatkova M.A., Pleshkova A.I., Fomenko K.A., Ustinov A.Y., Sinebryukhov S.L., Gnedenkov S.V. (2024). Effect of Ta_2_O_5_ Nanoparticles on Bioactivity, Composition, Structure, In Vitro and In Vivo Behavior of PEO Coatings on Mg-Alloy. J. Magnes. Alloys.

[3] McNamara K., Kolaj-Robin O., Belochapkine S., Laffir F., Gandhi A.A., Tofail S.A.M. (2015). Surface Chemistry and Cytotoxicity of Reactively Sputtered Tantalum Oxide Films on NiTi Plates. Thin Solid Films.

[4] Asadullah S., Ahmed M., Sarfraz S., Zahra M., Asari A., Wahab N.H.A., Sobia F., Iqbal D.N. (2023). Polyimide Biocomposites Coated with Tantalum Pentoxide for Stimulation of Cell Compatibility and Enhancement of Biointegration for Orthopedic Implant. Heliyon.

[5] Song G., Chao Y., Chen Y., Liang C., Yi X., Yang G., Yang K., Cheng L., Zhang Q., Liu Z. (2016). All-in-One Theranostic Nanoplatform Based on Hollow TaOx for Chelator-Free Labeling Imaging, Drug Delivery, and Synergistically Enhanced Radiotherapy. Adv. Funct. Mater..

[6] Kant R., Tabassum R., Gupta B.D. (2017). A Highly Sensitive and Distinctly Selective D-Sorbitol Biosensor Using SDH Enzyme Entrapped Ta_2_O_5_ Nanoflowers Assembly Coupled with Fiber Optic SPR. Sens. Actuators B Chem..

[7] Yeh B.M., FitzGerald P.F., Edic P.M., Lambert J.W., Colborn R.E., Marino M.E., Evans P.M., Roberts J.C., Wang Z.J., Wong M.J. (2017). Opportunities for New CT Contrast Agents to Maximize the Diagnostic Potential of Emerging Spectral CT Technologies. Adv. Drug Deliv. Rev..

[8] Chakravarty S., Hix J.M.L., Wiewiora K.A., Volk M.C., Kenyon E., Shuboni-Mulligan D.D., Blanco-Fernandez B., Kiupel M., Thomas J., Sempere L.F. (2020). Tantalum Oxide Nanoparticles as Versatile Contrast Agents for X-Ray Computed Tomography. Nanoscale.

[9] Peng C., Liang Y., Chen Y., Qian X., Luo W., Chen S., Zhang S., Dan Q., Zhang L., Li M. (2020). Hollow Mesoporous Tantalum Oxide Based Nanospheres for Triple Sensitization of Radiotherapy. ACS Appl. Mater. Interfaces.

[10] Kolyvanova M.A., Belousov A.V., Krusanov G.A., Isagulieva A.K., Morozov K.V., Kartseva M.E., Salpagarov M.H., Krivoshapkin P.V., Dement’eva O.V., Rudoy V.M. (2021). Impact of the Spectral Composition of Kilovoltage X-Rays on High-Z Nanoparticle-Assisted Dose Enhancement. Int. J. Mol. Sci..

[11] Gustafson H.H., Holt-Casper D., Grainger D.W., Ghandehari H. (2015). Nanoparticle Uptake: The Phagocyte Problem. Nano Today.

[12] Ernst L., Casals E., Italiani P., Boraschi D., Puntes V. (2021). The Interactions between Nanoparticles and the Innate Immune System from a Nanotechnologist Perspective. Nanomaterials.

[13] Moore T.L., Rodriguez-Lorenzo L., Hirsch V., Balog S., Urban D., Jud C., Rothen-Rutishauser B., Lattuada M., Petri-Fink A. (2015). Nanoparticle Colloidal Stability in Cell Culture Media and Impact on Cellular Interactions. Chem. Soc. Rev..

[14] Wang R., Zhang Z., Liu B., Xue J., Liu F., Tang T., Liu W., Feng F., Qu W. (2021). Strategies for the Design of Nanoparticles: Starting with Long-Circulating Nanoparticles, from Lab to Clinic. Biomater. Sci..

[15] Bozzer S., Grimaldi M.C., De Maso L., Manfredi M., Toffoli G., Dal Bo M., Sblattero D., Macor P. (2024). Stealth-Engineered Albumin-Coated Nanoparticles for Targeted Therapy: Effective Drug Delivery and Tumor Suppression in Xenograft-Zebrafish Model. Int. J. Nanomed..

[16] Gan N., Sun Q., Zhao L., Tang P., Suo Z., Zhang S., Zhang Y., Zhang M., Wang W., Li H. (2019). Protein Corona of Metal-Organic Framework Nanoparticals: Study on the Adsorption Behavior of Protein and Cell Interaction. Int. J. Biol. Macromol..

[17] Žūkienė R., Snitka V. (2015). Zinc Oxide Nanoparticle and Bovine Serum Albumin Interaction and Nanoparticles Influence on Cytotoxicity In Vitro. Colloids Surf. B Biointerfaces.

[18] Ali M., Nasir S., Ensinger W. (2016). Stereoselective Detection of Amino Acids with Protein-Modified Single Asymmetric Nanopores. Electrochim. Acta.

[19] Zhu L.-P., Jiang J.-H., Zhu B.-K., Xu Y.-Y. (2011). Immobilization of Bovine Serum Albumin onto Porous Polyethylene Membranes Using Strongly Attached Polydopamine as a Spacer. Colloids Surf. B Biointerfaces.

[20] Li H., He E., Wang Y., Fu J., Liu T., Gou R., Shi S., Gu C. (2023). Albumin-Stabilized Polydopamine Nanoparticles for Chemo-Photothermal Synergistic Therapy of Melanoma. J. Drug Deliv. Sci. Technol..

[21] Silva-Bermudez P., Rodil S.E., Muhl S. (2011). Albumin Adsorption on Oxide Thin Films Studied by Spectroscopic Ellipsometry. Appl. Surf. Sci..

[22] Liu G., Wu C., Zhang X., Liu Y., Meng H., Xu J., Han Y., Xu X., Xu Y. (2016). Surface Functionalization of Zirconium Dioxide Nano-Adsorbents with 3-Aminopropyl Triethoxysilane and Promoted Adsorption Activity for Bovine Serum Albumin. Mater. Chem. Phys..

[23] Bukackova M., Marsalek R. (2020). Interaction of BSA with ZnO, TiO_2_, and CeO_2_ Nanoparticles. Biophys. Chem..

[24] Goodilin E.A., Weiss P.S., Gogotsi Y. (2019). Nanotechnology Facets of the Periodic Table of Elements. ACS Nano.

[25] Stordy B.P., Sepahi Z., Patrón G.D., Yang W., Goodson A.D., Blackadar C., Tavares A.J., Lin G., Malekjahani A., Ling B. (2025). The Binding Affinities of Serum Proteins to Nanoparticles. J. Am. Chem. Soc..

[26] Koshevaya E., Mikhaylov V., Sitnikov P., Krivoshapkina E., Krivoshapkin P. (2022). Electrosurface Properties and Acid-Base Equilibria of Ta_2_O_5_ and Ta_2_O_5_:Eu Nanoparticles in NaCl Solutions. Surf. Interfaces.

[27] Koshevaya E., Nazarovskaia D., Simakov M., Belousov A., Morozov V., Gandalipov E., Krivoshapkina E., Krivoshapkin P. (2020). Surfactant-Free Tantalum Oxide Nanoparticles: Synthesis, Colloidal Properties, and Application as a Contrast Agent for Computed Tomography. J. Mater. Chem. B.

[28] Lu M., Yu J., Chun H.J., Park K., Kim C.-H., Khang G. (2018). Mussel-Inspired Biomaterials for Cell and Tissue Engineering. Novel Biomaterials for Regenerative Medicine.

[29] Ye Q., Zhou F., Liu W. (2011). Bioinspired Catecholic Chemistry for Surface Modification. Chem. Soc. Rev..

[30] Koshevaya E.D., Shishmakova E.M., Belousov A.V., Morozov V.N., Kolyvanova M.A., Dement’eva O.V. (2025). Synthesis of hybrid Ta_2_O_5_@PDA/Au nanocomposites. Mendeleev Commun..

[31] Lin J.-H., Yu C.-J., Yang Y.-C., Tseng W.-L. (2015). Formation of Fluorescent Polydopamine Dots from Hydroxyl Radical-Induced Degradation of Polydopamine Nanoparticles. Phys. Chem. Chem. Phys..

[32] Sy K.H.S., Ho L.W.C., Lau W.C.Y., Ko H., Choi C.H.J. (2018). Morphological Diversity, Protein Adsorption, and Cellular Uptake of Polydopamine-Coated Gold Nanoparticles. Langmuir.

[33] Starosta R., Santos F.C., De Almeida R.F.M. (2020). Human and Bovine Serum Albumin Time-Resolved Fluorescence: Tryptophan and Tyrosine Contributions, Effect of DMSO and Rotational Diffusion. J. Mol. Struct..

[34] Shekhar H., Behera P., Naik A., Mishra M., Sahoo H. (2024). Interaction between Polydopamine-Based IONPs and Human Serum Albumin (HSA): A Spectroscopic Analysis with Cytotoxicity Impact. Nanotoxicology.

[35] Lakowicz J.R. (2006). Principles of Fluorescence Spectroscopy.

[36] Kumar Panigrahi S., Kumar Mishra A. (2019). Inner Filter Effect in Fluorescence Spectroscopy: As a Problem and as a Solution. J. Photochem. Photobiol. C Photochem. Rev..

[37] Barbero N., Barni E., Barolo C., Quagliotto P., Viscardi G., Napione L., Pavan S., Bussolino F. (2009). A Study of the Interaction between Fluorescein Sodium Salt and Bovine Serum Albumin by Steady-State Fluorescence. Dyes Pigment..

[38] Hao C., Xu G., Feng Y., Lu L., Sun W., Sun R. (2017). Fluorescence Quenching Study on the Interaction of Ferroferric Oxide Nanoparticles with Bovine Serum Albumin. Spectrochim. Acta A Mol. Biomol. Spectrosc..

[39] Sun W., Du Y., Chen J., Kou J., Yu B. (2009). Interaction between Titanium Dioxide Nanoparticles and Human Serum Albumin Revealed by Fluorescence Spectroscopy in the Absence of Photoactivation. J. Lumin..

[40] Tsykhanovska I., Stabnikova O., Gubsky S. Spectroscopic Studies of Interactions of Iron Oxide Nanoparticles with Ovalbumin Molecules. Proceedings of the 3rd International Online-Conference on Nanomaterials.

[41] Yang Q., Liang J., Han H. (2009). Probing the Interaction of Magnetic Iron Oxide Nanoparticles with Bovine Serum Albumin by Spectroscopic Techniques. J. Phys. Chem. B.

[42] Ning J., Zhang J., Suo T., Yin Z. (2018). Spectroscopic Studies of Human Serum Albumin Exposed to Fe_3_O_4_ Magnetic Nanoparticles Coated with Sodium Oleate: Secondary and Tertiary Structure, Fibrillation, and Important Functional Properties. J. Mol. Struct..

[43] Ghosh D., Chattopadhyay N. (2015). Gold and Silver Nanoparticles Based Superquenching of Fluorescence: A Review. J. Lumin..

[44] Hao N., Lv R., Gao S., Yan Y., Gao X., Tian R., Lv R., Zhu K., Shi G., Ji Y. (2025). Insights on the Interactions between Functionalized Metal-Polyphenol Nanocarriers and Human Serum Albumin via Spectroscopic Analysis. J. Drug Deliv. Sci. Technol..

[45] Htun T. (2004). A Negative Deviation from Stern–Volmer Equation in Fluorescence Quenching. J. Fluoresc..

[46] Wang Y., Jiang Q., Liu L.R., Zhang Q. (2007). The Interaction between Bovine Serum Albumin and the Self-Aggregated Nanoparticles of Cholesterol-Modified O-Carboxymethyl Chitosan. Polymer.

[47] Valeur B., Berberan-Santos M.N. (2012). Molecular Fluorescence: Principles and Applications.

[48] Cornish-Bowden A. (2013). Fundamentals of Enzyme Kinetics.

[49] Motulsky H.J., Ransnas L.A. (1987). Fitting Curves to Data Using Nonlinear Regression: A Practical and Nonmathematical Review. FASEB J..

[50] Naik A.B., Naik L.R., Kadadevarmath J.S., Pal H., Rao V.J. (2010). Fluorescence Quenching of Anthrylvinyl Acetate by Carbon Tetrachloride. J. Photochem. Photobiol. Chem..

[51] Wei X.L., Xiao J.B., Wang Y., Bai Y. (2010). Which Model Based on Fluorescence Quenching Is Suitable to Study the Interaction between Trans-Resveratrol and BSA?. Spectrochim. Acta A Mol. Biomol. Spectrosc..

[52] Van De Weert M., Stella L. (2011). Fluorescence Quenching and Ligand Binding: A Critical Discussion of a Popular Methodology. J. Mol. Struct..

[53] Sousa A.A. (2015). A Note on the Use of Steady–State Fluorescence Quenching to Quantify Nanoparticle–Protein Interactions. J. Fluoresc..

[54] Goutelle S., Maurin M., Rougier F., Barbaut X., Bourguignon L., Ducher M., Maire P. (2008). The Hill Equation: A Review of Its Capabilities in Pharmacological Modelling. Fundam. Clin. Pharmacol..

[55] Yang J.A., Johnson B.J., Wu S., Woods W.S., George J.M., Murphy C.J. (2013). Study of Wild-Type α-Synuclein Binding and Orientation on Gold Nanoparticles. Langmuir.

[56] Mariam J., Dongre P.M., Kothari D.C. (2011). Study of Interaction of Silver Nanoparticles with Bovine Serum Albumin Using Fluorescence Spectroscopy. J. Fluoresc..

[57] Gesztelyi R., Zsuga J., Kemeny-Beke A., Varga B., Juhasz B., Tosaki A. (2012). The Hill Equation and the Origin of Quantitative Pharmacology. Arch. Hist. Exact Sci..

[58] Bisswanger H. (2008). Enzyme Kinetics: Principles and Methods.

[59] Philippova O.E., Korchagina E.V. (2012). Chitosan and Its Hydrophobic Derivatives: Preparation and Aggregation in Dilute Aqueous Solutions. Polym. Sci. Ser. A.

[60] Tayeh N., Rungassamy T., Albani J.R. (2009). Fluorescence Spectral Resolution of Tryptophan Residues in Bovine and Human Serum Albumins. J. Pharm. Biomed. Anal..

[61] Greenfield N.J. (1996). Methods to Estimate the Conformation of Proteins and Polypeptides from Circular Dichroism Data. Anal. Biochem..

[62] Banu A., Naqvi S., Qashqoosh M.T.A., Kadaf Manea Y., Laiq E. (2023). Synthesis and Characterization of Bisacodyl Loaded Chitosan Nanoparticles (BSL@CS NPs), Multispectroscopic Study of Their Interaction with Bovine Serum Albumin (BSA). J. Mol. Liq..

[63] Liu J., Tian J., Tian X., Hu Z., Chen X. (2004). Interaction of Isofraxidin with Human Serum Albumin. Bioorg. Med. Chem..

[64] Rogozea A., Matei I., Turcu I.M., Ionita G., Sahini V.E., Salifoglou A. (2012). EPR and Circular Dichroism Solution Studies on the Interactions of Bovine Serum Albumin with Ionic Surfactants and β-Cyclodextrin. J. Phys. Chem. B.

[65] Peters T. (1996). All About Albumin: Biochemistry, Genetics, and Medical Applications.

[66] Gharagozlou M., Boghaei D.M. (2008). Interaction of Water-Soluble Amino Acid Schiff Base Complexes with Bovine Serum Albumin: Fluorescence and Circular Dichroism Studies. Spectrochim. Acta A Mol. Biomol. Spectrosc..

[67] Anand U., Mukherjee S. (2013). Reversibility in Protein Folding: Effect of β-Cyclodextrin on Bovine Serum Albumin Unfolded by Sodium Dodecyl Sulphate. Phys. Chem. Chem. Phys..

[68] Dominguez-Medina S., Kisley L., Tauzin L.J., Hoggard A., Shuang B., Indrasekara A.S.D.S., Chen S., Wang L.-Y., Derry P.J., Liopo A. (2016). Adsorption and Unfolding of a Single Protein Triggers Nanoparticle Aggregation. ACS Nano.

[69] Li S., Peng Z., Leblanc R.M. (2015). Method To Determine Protein Concentration in the Protein–Nanoparticle Conjugates Aqueous Solution Using Circular Dichroism Spectroscopy. Anal. Chem..

[70] Bellamy L.J. (1975). The Infra-Red Spectra of Complex Molecules.

[71] Li X., Guo M., Xie C., Xue Y., Zhang J., Zhang D., Duan Z. (2025). Interaction Between Bovine Serum Albumin and Trans-Resveratrol: Multispectroscopic Approaches and Molecular Dynamics Simulation. Foods.

[72] Mrówczyński R., Jurga-Stopa J., Markiewicz R., Coy E.L., Jurga S., Woźniak A. (2016). Assessment of Polydopamine Coated Magnetic Nanoparticles in Doxorubicin Delivery. RSC Adv..

[73] Li Y., Xu H., Li H., Zhong S. (2021). Controlled Preparation and Photothermal Properties of Polydopamine Submicrospheres. Inorg. Chem. Commun..

[74] Dement’eva O.V., Naumova K.A., Shishmakova E.M., Senchikhin I.N., Zhigletsova S.K., Klykova M.V., Dunaitsev I.A., Kozlov D.A., Rudoy V.M. (2021). Synthesis of Bifunctional Silica Container Particles on Antiseptic Micelles with Solubilized Curcumin and Assessment of Their Biological Activity. Colloid J..

[75] Koshevaya E.D., Maslov D.D., Shishmakova E.M., Mikhaylov V.I., Belousov A.V., Grafov O.Y., Vodyashkin A.A., Morozov V.N., Kolyvanova M.A., Dement’eva O.V. (2026). Hybrid Ta_2_O_5_-Au Nanoparticles Synthesized by Radiolytic Reduction of Gold Ions: Effects of Synthesis Parameters and Tantalum Oxide Surface Chemistry. Colloids Surf. Physicochem. Eng. Asp..

