# Supplementary material for: Interaction of BSA with Ta2O5 Nanoparticles: The Effect of Polydopamine Pre-Coating
Source: Molecules. 2026 Jan 11;31(2):241. doi: 10.3390/molecules31020241 (PMC12843971; doi:10.3390/molecules31020241)
Supplement: Supplementary file 1 [file molecules-31-00241-s001.zip › molecules-4034401-supplementary.pdf]

Supplementary information

Interaction of BSA with Ta_2O_5 Nanoparticles: The Effect of Polydopamine Pre-Coating

Ekaterina Koshevaya [1,*], Nikita Lifanovsky [2,3], Elena Shishmakova [4], Maksim Staltsov [3], Alexander Dubovik [2], Alexandr Belousov [1], Dmitry Kaluzhny [5], Vladimir Kuzmin [2], Vladimir Morozov [2], Maria Kolyvanova [1,2] and Olga Dement'eva [4]

[1] State Research Center—Burnazyan Federal Medical Biophysical Center, Federal Medical Biological Agency of the Russian Federation, 23 Marshala Novikova, Moscow 123182, Russia;

[2] Emanuel Institute of Biochemical Physics, Russian Academy of Sciences, 4 Kosygina, Moscow 119334, Russia;
[3] National Research Nuclear University MEPhI, 31 Kashirskoye Shosse, Moscow 115409, Russia;
[4] Frumkin Institute of Physical Chemistry and Electrochemistry, Russian Academy of Sciences, 31-4 Leninsky Prospect, Moscow 119071, Russia;
[5] Engelhardt Institute of Molecular Biology, Russian Academy of Sciences, 32 Vavilova, Moscow 119991, Russia; * Correspondence: katiakosh@gmail.com

Table S1. Hydrodynamic diameters (Z-average) and polydispersity index (in brackets) of NPs in water and PBS. Values are presented as mean ± standard deviation of at least three measurements.

Sample	Water	PBS (pH=7.4)
Ta_2O_5	70.4 ± 0.3 (0.235)	129.1 ± 0.7 (0.191)
Ta_2O_5-BSA	72.0 ± 0.7 (0.179)	80.6 ± 0.7 (0.284)
Ta_2O_5@PDA	86.5 ± 0.4 (0.104)	959.3 ± 40.0 (0.262)
Ta_2O_5@PDA-BSA	89.1 ± 0.4 (0.101)	91.4 ± 0.4 (0.100)

The FTIR spectra of Ta_2O_5, Ta_2O_5@PDA, Ta_2O_5-BSA, and Ta_2O_5@PDA-BSA NPs are presented on Fig. S1 (full spectra (a) and their elongated parts (b)). The FTIR spectrum of Ta_2O_5 NPs (black) exhibits a broad peak (500–1000 cm^{-1}) attributed to vibrations in the metal oxide lattice, alongside characteristic O–H stretching vibrations (3420 cm^{-1}) and H_2O deformation vibrations (1630 cm^{-1}). For Ta_2O_5@PDA NPs (dark blue), a series of new peaks emerges in the range of 1250–1550 cm^{-1} is attributed to PDA coating [S1]. In Ta_2O_5-BSA (grey) and Ta_2O_5@PDA-BSA NPs (blue), amide bond formation is confirmed by the intensified absorption

band at 1648 cm^{-1}, corresponding to the C=O stretching vibrations (amide I) [S2]. Detailed spectral assignment remains challenging due to overlapping absorption bands from the protein (BSA), water and PDA.

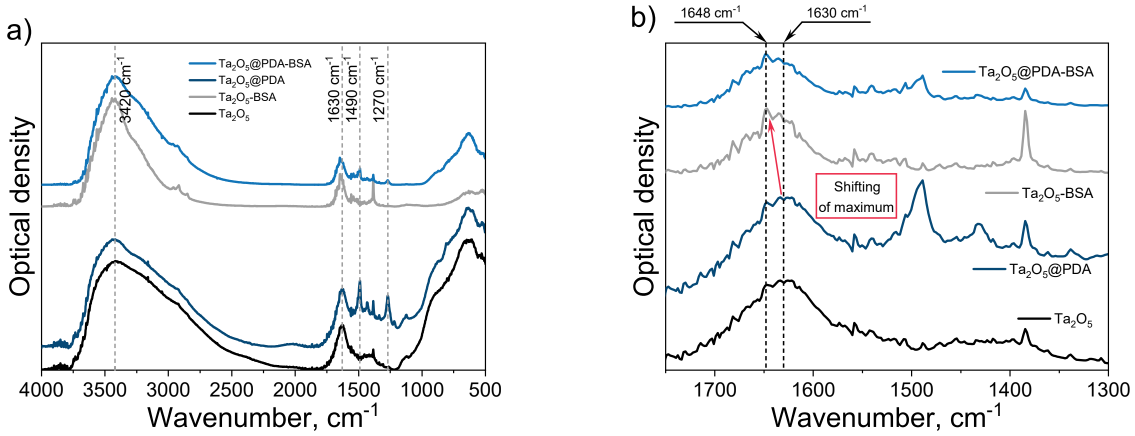

Figure S1 FTIR spectra of Ta_2O_5 (black), Ta_2O_5@PDA (dark blue), Ta_2O_5-BSA (grey) and Ta_2O_5@PDA-BSA NPs (blue) in the regions 500–4000 cm^{-1} (a) and 1300–1750 cm^{-1} (b).

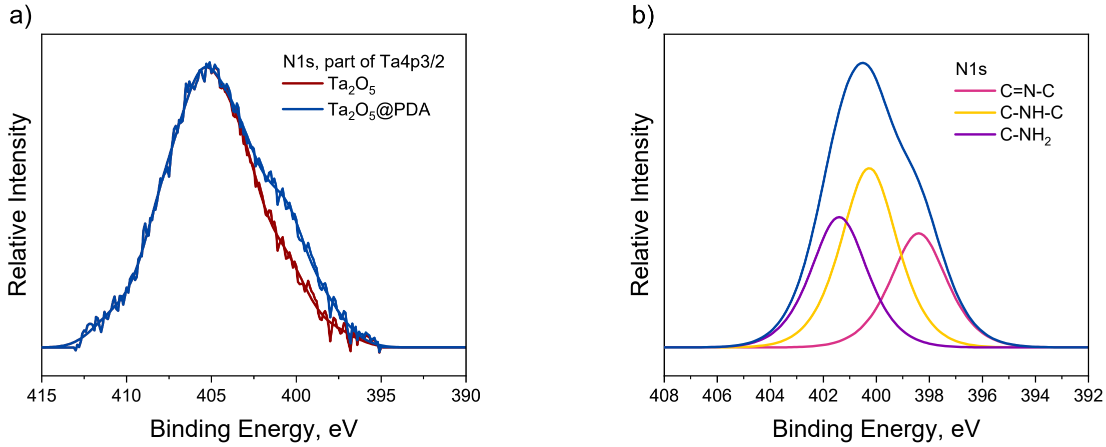

Figure S2 XPS spectra in the N1s region for bare Ta_2O_5 NPs (burgundy line) and Ta_2O_5@PDA NPs (dark blue line). Panel (a) shows the overlaid raw spectra, with the N1s peak of Ta_2O_5@PDA overlapping with the Ta $4p_{3/2}$ signal of bare Ta_2O_5. Panel (b) presents the deconvoluted N1s spectrum after subtraction of the Ta $4p_{3/2}$ contribution, showing the individual nitrogen bonding states.

Compared with Ta_2O_5 reference sample, the N1s XPS spectrum of Ta_2O_5@PDA NPs reveals a prominent absorption feature in the 395–405 eV binding energy range, characteristic of nitrogen-containing functional groups in the PDA (Fig. S2a). In the raw spectrum (Fig. S2a), the N1s peak of Ta_2O_5@PDA overlaps with the Ta $4p_{3/2}$ photoelectron line of bare Ta_2O_5. To isolate and analyze the nitrogen bonding states, the Ta $4p_{3/2}$ contribution from the bare Ta_2O_5 spectrum was subtracted from the Ta_2O_5@PDA data, yielding the deconvoluted N1s spectrum shown in

Fig. S2b. According to the peak positions, the following states can be distinguished: C=N-C (398.3 eV), C-NH-C (400.0 eV), C-NH_2 (401.5 eV).

References

S1. Koshevaya, E. D.; Shishmakova, E. M.; Belousov, A. V.; Morozov, V. N.; Kolyvanova, M. A.; Dement'eva, O. V. Synthesis of hybrid Ta_2O_5@PDA/Au nanocomposites. *Mendeleev Commun.* **2025**, *35* (4), 475–477. https://doi.org/10.71267/mencom.7672.

1. S2. Bellamy, L.J. *The Infra-Red Spectra of Complex Molecules*; Springer: Dordrecht, The Netherlands, 1975; ISBN 978-94-011-6019-3.
